# Changes in Physical Activity during COVID-19 Pandemic in Urban and Rural Older Veterans and their Cohabitants

**DOI:** 10.1093/geroni/igaa057.3428

**Published:** 2020-12-16

**Authors:** Chao-Yi Wu, Rachel Wall, Zachary Beattie, Nora Mattek, Jeffrey Kaye, Hiroko Dodge, Lisa Silbert

**Affiliations:** 1 Oregon Health & Science University, Portland, Oregon, United States; 2 Portland Veterans Affairs Medical Center, Portland, Oregon, United States; 3 Layton Alzheimer’s Disease Center, Portland, Oregon, United States; 4 Pregon Health & Science University, Portland, Oregon, United States

## Abstract

Background: The COVID-19 pandemic has changed the health security of older adults. Few have examined how older US veterans have reacted and coped with the COVID-19 pandemic. We aimed to identify changes in physical health and their differential impact by rurality of older veterans. Method: Participants were veterans (aged ≥ 62 years) and their cohabitants, living in the Pacific Northwest, enrolled in the Collaborative Aging Research using Technology (CART) initiative. Daily step counts via actigraphy were collected from January 1st to July 8th, 2020. COVID-19 time periods were determined by stay-at-home orders issued on March 13th, 2020. Generalized estimating equation models were used to examine changes in physical activities associated with COVID-19 time periods and rurality indicated by the rural-urban commuting area score. Results: A total of 102 participants were included in the analysis (mean age = 71.0 years, 56% male, 32 living in urban areas). Daily average step counts were 2318 and 3012 before and after COVID-19 stay-at-home orders (t=4.85, p<.001). After controlling for covariates, participants living in large rural (β=.26, p=.03) and small/isolated areas (β=.23, p=.02) walked more than those living in urban areas after COVID-19 stay-at-home orders. Conclusion: Older adults cope differently during the COVID-19 pandemic based on rurality, with those living in large rural and small/isolated rural areas have an increased physical activity. Reasons for increased step counts (e.g., mood, visitors, size of the house) require further investigation. This result demonstrates the potential utility of real-world monitoring to objectively inform interventions for COVID-related secondary health changes.

